# Experimental Comparison of IR-UWB Radar and FMCW Radar for Vital Signs

**DOI:** 10.3390/s20226695

**Published:** 2020-11-23

**Authors:** Dingyang Wang, Sungwon Yoo, Sung Ho Cho

**Affiliations:** Department of Electronics and Computer Engineering, Hanyang University, Seoul 04763, Korea; wdyggh@hanyang.ac.kr (D.W.); irishtaco@hanyang.ac.kr (S.Y.)

**Keywords:** impulse radio ultra-wideband radar, FMCW radar, vital signs, noncontact measurement

## Abstract

In this paper, we compare the performances of impulse radio ultra-wideband (IR-UWB) and frequency modulation continuous wave (FMCW) radars in measuring noncontact vital signs such as respiration rate and heart rate. These two type radars have been widely used in various fields and have shown their applicability to extract vital signs in noncontact ways. IR-UWB radar can extract vital signs using distance information. On the other hand, FMCW radar requires phase information to estimate vital signs, and the result can be enhanced with Multi-input Multi-output (MIMO) antenna topologies. By using commercial radar chipsets, the operation of radars under different conditions and frequency bands will also affect the performance of vital sign detection capabilities. We compared the accuracy and signal-to-noise (SNR) ratios of IR-UWB and FMCW radars in various scenarios, such as distance, orientation, carotid pulse, harmonics, and obstacle penetration. In general, the IR-UWB radars offer a slightly better accuracy and higher SNR in comparison to FMCW radar. However, each radar system has its own unique advantages, with IR-UWB exhibiting fewer harmonics and a higher SNR, while FMCW can combine the results from each channel.

## 1. Introduction

Respiratory rate (RR) and heart rate (HR) are key physiological parameters in monitoring human vital signs. Traditional contact measurement techniques include respiration belt, electrocardiogram (ECG), and photoplethysmography (PPG). These contact measurement methods may cause discomfort, disconnection due to cable kinks, and epidermal stripping, such as in monitoring infants, patient with severe skin burns, or sleep. There is a need to develop accurate and noncontact vital sign detection and monitoring methods that can be used in a variety of scenarios including sleep quality monitoring, daily healthcare, fall detection, and even elderly monitoring.

A camera [1,2] can be can be used to monitor noncontact vital signs by measuring changes in skin color that are the result of changes in blood volume, but the accuracy can be affected by ambient lighting; when the light illuminance decreases, the accuracy of the camera decreases significantly. This means that even if the optical technique can measure blood oxygen saturation, it cannot be used in a dark environment or at night. Additionally, light cannot penetrate objects such as bricks and concrete, which limits its use in rescue operations. The use of cameras may also raise privacy concerns.

Another noncontact measuring technique is radar. Radar is used to measure the distance of an object by transmitting microwaves. Due to advantages such as the high resolution, phase information, or MIMO antenna topologies of the radar, there has been an increase in research on the use of radar to measure vital signs [3,4,5,6,7,8]. For example, IR-UWB radar has been implemented for detecting vital signs during rescue operations [9,10], and FMCW radar has been used to detect infant or animal occupants in vehicles [11] and in advanced driver-assistance systems (ADAS) [12,13,14].

IR-UWB radar is widely used in several wireless sensing scenarios such as to count people [15], detect humans through walls [16], and monitor home environments [17]. Studies have also been conducted on measuring vital signs [18] and distinguishing humans from animals by vital signs [19]. IR-UWB radar transmits a short gaussian pulse without a carrier frequency in the time domain, which has a wide band in the frequency domain. The power of the pulse is limited and meets the Federal Communications Frequency mask [20]. In order to distinguish the target from the noise, IR-UWB radar needs to transmit multiple pulses to increase the signal-to-noise ratio. IR-UWB radar also requires a high-speed analog-to-digital converter (ADC), which may increase the cost and complexity of hardware design.

FMCW radar overcomes the drawbacks of continuous-wave (CW) radar, with capabilities to measure range and velocity, which has also been studied in measuring noncontact vital signs [21,22] as well as counting people and recognizing hand gestures. Since it transmits linear FM signals, it can transmit relatively high power to achieve a high SNR. However, it still requires calibration to compensate for nonlinearities during frequency sweeping [23,24]. Compared to IR-UWB radar, FMCW uses low-speed ADC because it has a narrow instantaneous bandwidth. In addition, MIMO antenna topologies are capable of localizing multiple targets [25] or monitoring in real time.

The algorithms for extracting vital signs vary due to operation principles. IR-UWB radar uses the pulse to measure range, and chest motion can be measured by the amplitude. FMCW uses chirp to measure the distance and velocity, and vital signs can be estimated with phase information. Due to the hardware configuration, MIMO topologies can be easily designed to estimate the direction of arrival (DOA). In the experimental comparison, we compared the accuracy and SNR ratio of RR and HR using two commercial radars and analyzed the vital signs detection capabilities.

In most studies, radar results are compared to a reference sensor. In this work, we used the respiration belt and ECG sensor as references. In [26], the ratio of the measured value to the reference sensor value is considered the accuracy of measurements; the ratios were 103% (i.e., +3% error for respiration) and 95% (i.e., −5% error for heart rate). The error could be related to body movement.

This paper is organized as follows. In Section 2, the operation principles of IR-UWB radar and FMCW radar as well as the basic algorithm to detect vital signs are described. In Section 3, the experimental environment is described, which is composed of IR-UWB radar, FMCW radar, and reference sensors to compare their performances. The experiment is performed in multiple design scenarios. In Section 4, we compare the performance of the radars in each scenario, and all results were compared to contact reference sensors. Finally, we discuss the advantages and limitations of each radar in detecting vital signs, and we conclude in Section 6.

## 2. IR-UWB and FMCW Principle

**Figure 1:**
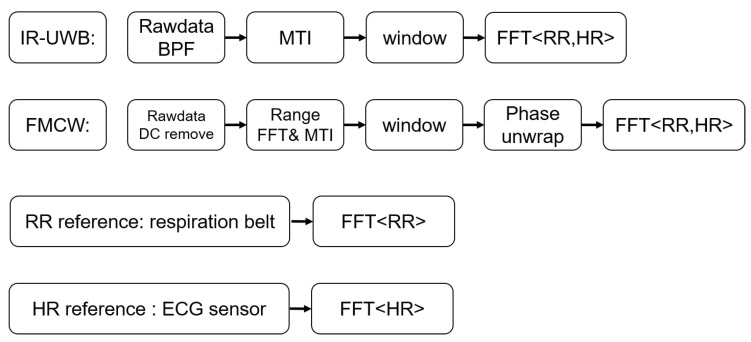
Block diagram of the algorithm used in comparison.

Generally, using radar to monitor vital signs involves measuring the torso displacement caused by respiration and heartbeat motions. IR-UWB uses the amplitude of an estimated human body point to extract the vital signals. On the other hand, FMCW radar unwraps the phase of an estimated human body point to estimate the respiration rate and heartbeat rate. A block diagram of the algorithm used is shown in Figure 1. The working principle for using IR-UWB radar and FMCW radar to monitor vital signs is described below.

### 2.1. IR-UWB Radar

IR-UWB radar transmits pulses and hits the human body. The time-of-arrival (ToA) of this pulse is denoted by $\tau_{0}$ and depends on the distance between the radar and subject $d_{0}$. The signal reflected from the subject’s body with movement caused by respiration and heartbeat can be written as

(1) $$\begin{array}{cc} d\left(t\right) & =d_{0}+m\left(t\right)\\ \; & =d_{0}+m_{b}sin\left(2\pi f_{b}t\right)+m_{h}sin\left(2\pi f_{h}t\right) \end{array}$$

where $m_{b}\;\mathrm{and}\;m_{h}$ are the amplitudes of respiration and heartbeat. $f_{b}\;\mathrm{and}\;f_{h}$ are the frequency of respiration and heartbeat.

The received signal can be represented as the sum of the channel’s response and variation due to respiration and heartbeat:

(2) $$r\left(t,\tau \right)=\sum_{i}A_{i}p\left(\tau -\tau_{i}\right)+Ap\left(\tau -\tau_{d}\left(t\right)\right)$$

$p\left(t\right)$ is the normalized received pulse. $A_{i}$ is the amplitude of each multipath component. $\tau_{i}$ is the delay of each multipath. *A* is the amplitude of the pulse-reflected of body.

The time delay $\tau_{d}$ with vital signs is modeled as the sum of the time-of-flight $\tau_{0}$ and two sinusoidal delays associated with respiration and heartbeat.

(3) $$\tau_{d}\left(t\right)=2d\left(t\right)/c=\tau_{0}+\tau_{b}sin\left(2\pi f_{b}t\right)+\tau_{h}sin\left(2\pi f_{h}t\right)$$

The received signal matrix $R_{IR}$ is measured at discrete instants in slow time $t=nT_{s}\;\left(n=1,2,3\ldots N\right)$ with sampling rate $T_{f}$ measured in the fast time.

(4) $$R_{IR}\left[n,m\right]=r\left(t=mT_{s},\tau =nT_{f}\right)$$

In a static environment, the resulting clutter can be considered as a DC-component in the slow-time direction. The clutter does not depend on slow time. Thus, static background clutter can be eliminated by filtering methods while maintaining the subject signal. The movement is only caused by respiration and heartbeat movements in the human. Let $r\left(t,\tau \right)$ represent the received signal, the DC-removed signal $x\left(t,\tau \right)$ can be written as

(5) $$x\left(t,\tau \right)=r\left(t,\tau \right)-r_{0}\left(\tau \right)$$

$r_{0}\left(\tau \right)$ is the DC component.

According to (3), the ideal signal of interest without a stationary background can be written as below:

(6) $$y\left(t,\tau \right)=Ap\left(\tau -\tau_{d}\left(t\right)\right)=Ap\left(\tau -\tau_{0}+\tau_{b}sin\left(2\pi f_{b}t\right)+\tau_{h}sin\left(2\pi f_{h}t\right)\right)$$

The IR-UWB radar is used with 1 transmit channel and 1 receive channel, and the received impulse signal data are saved in matrix $R_{IR}$. The algorithm diagram used to extract the vital signs using IR-UWB radar is shown in Figure 1.

1. Due to the short impulse in the time-domain, the received signal has a large bandwidth. A bandpass filter is used over each row of the raw ADC data matrix $R_{IR}$, resulting in time-domain matrix $D_{IR}\;\left[n,m\right]$, where m=1,2,…,M denotes the fast-time samples in the range direction, and n=1,2,…,N denotes the index of received frames in the slow-time dimension.
2. In order to extract the human body signal from the raw data signal with background noise and stationary clutter, the recursive filter [20] is one of the MTI algorithms used for this situation. The MTI filter generates the clutter-suppressed signal $D_{IR-MTI}\;\left[n,m\right]$ by subtracting the estimated clutter from the received raw data signal.
  (7) $$C\;\left[n,m\right]=D_{IR-MTI}\;\left[n-1,m\right]+C\;\left[n-1,m\right]$$
  (8) $$D_{IR-MTI}\;\left[n,m\right]=D_{IR}\;\left[n,m\right]-\left(1-\alpha \right)C\;\left[n,m\right]$$
  where $C\;\left[n,m\right]$ represents the estimated clutter signal at the *n*-th slow time. α is the gain factor one can control in the filter response. If the gain factor is set to be high, the amplitude of the subject is high in the clutter removal signal, but it takes more time to remove the clutter components.
3. The range bin of the human body is determined for each row of $D_{IR-MTI}\;\left[n,m\right]$ by finding the maximum variance point within a slow-time slide window, and values for each frame are saved as $V_{IR}\;\left[n\right]$. $V_{IR}\;\left[n\right]$ is the vital signs extracted from the raw data and consists of respiration and heart beat.
4. Fast fourier transform (FFT) spectral analysis is applied to $V_{IR}\;\left[n\right]$ for respiration rate and heartbeat rate.

**Figure 2:**
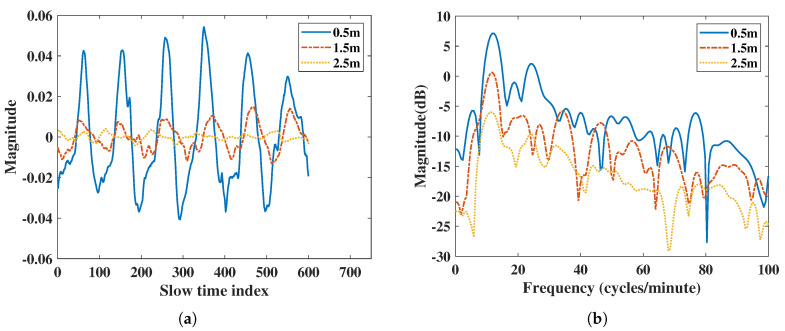
Vital signs extracted using IR-UWB radar at 0.5 m, 1.5 m, and 2.5 m: (**a**) vital signs extracted by IR-UWB radar in the time domain, (**b**) vital signs extracted by IR-UWB radar in the frequency domain.

The algorithm used in IR-UWB estimates the position of the subject’s body and saves its amplitude value as the respiration and heartbeat movement in the range direction. According to the radar power equation [27], the power of microwave signal is attenuated 4-fold by the distance product. From the vital signs result in Figure 2 extracted using IR-UWB radar, the result shows a significantly different amplitude due to attenuation when the subject is at different distances. Power and SNR are regraded to the amplitude of the vital signals. The power of RR or HR decreases due to different amplitude. In order to do a fair comparison of power and SNR with IR-UWB and FMCW radar at different distances, the normalized vital signs signal at the same range is used when performing the FFT spectral analysis.

### 2.2. FMCW Radar

The FMCW signal models periodic, linearly increasing frequency ramps and are transmitted as follows:

(9) $$x_{T}\;\left(t\right)\;=\;A_{T}cos\;\left(2\pi f_{c}t\;+\;\pi \frac{B}{T_{c}}t^{2}\;+\;\phi \;\left(t\right)\right)$$

$A_{T}$ is transmitted power. $\phi \;\left(t\right)$ is the phase noise. $f_{c}$ is the chirp start frequency. *B* is the bandwidth of the chirp. $T_{C}$ is the duration of the chirp.

The signal at the receiver was scaled and shifted $x_{T\left(t\right)}$ and can be described as

(10) $$x_{R}\;\left(t\right)\;=\;\alpha x_{T}\;\left(t-t_{d}\right)\;=\;\alpha A_{T}cos\;\left(2\pi f_{c}\;\left(t\;-\;t_{d}\right)\;+\;\pi \frac{B}{T_{c}}\;{\left(t\;-\;t_{d}\right)}^{2}\;+\;\phi \;\left(t\;-\;t_{d}\right)\right)$$

α is a scale factor that denotes the attenuation of propagation, and $t_{d}\;=\;2R\left(t\right)/c$ is the range-dependent time delay from a given object at radial range $R\left(t\right)$. The received signal is then mixed with a replica of the transmitted signal. After I/Q mixing, the signal can be approximated as

(11) $$\begin{array}{cc} y\left(t\right) & =\;A_{R}e^{j\left(2\pi \left[\frac{B}{T_{c}}t_{d}\right]t+2\pi f_{c}t_{d}\;+\;\pi \frac{B}{T_{c}}t_{d}^{2}\;+\;\Delta \phi \left(t\right)\right)}\\ \; & =\;A_{R}e^{j\left(2\pi f_{b}t\;+\;\phi_{b}\left(t\right)\;+\;\Delta \phi \left(t\right)\right)} \end{array}$$

$A_{R}$ is the received signal power. $\Delta \phi \left(t\right)$ is the residual phase noise and can be neglected for short-range radar applications due to the range-correlation effect. $f_{b}$ is the beat frequency given by

(12) $$f_{b}\;=\;\frac{2BR\;\left(t\right)}{cT_{c}}$$

$\phi_{b}\;\left(t\right)$ is the phase of the received signal.

(13) $$\phi_{b}\;\left(t\right)\;=\;2\pi f_{c}t_{d}\;+\;\pi \frac{B}{T_{c}}t_{d}^{2}$$

The beat signal after I/Q sampling can be expressed for the nth ADC sample and mth chirp as

(14) $$y\;\left[n,m\right]\;=\;A_{R}e^{j\left(2\pi f_{b}nT_{f}\;+\;\frac{4\pi }{\lambda }R\left(nT_{f}\;+\;mT_{s}\right)\right)}$$

$T_{f}$ is the Fast-time ADC sampling interval. $T_{s}$ is the Slow-time sampling interval. The body surface movement caused by vital signs is small and has a low frequency. This implies that there would be no significant phase changes during the chirp time. Therefore, it is sufficient to measure the phase changes caused between successive chirps in slow time. Assume if the subject is at range $\left(R_{l}\right)$, based on the above equation, the phase shift will be

(15) $$\phi_{b}\;\left(nT_{f}\;+\;mT_{s}\right)\;=\;\frac{4\pi R_{l}\;\left(nT_{f}\;+\;mT_{s}\right)}{\lambda }$$

Suppose the FMCW radar used 1 transmit channel and 1 receive channel, then the received immediate signal data are saved in matrix $R_{FMCW}$.

1. Inverse fast fourier transform (IFFT) is performed over each row of the raw ADC data matrix $R_{FMCW}$. The result is the time-domain matrix $D_{FMCW}\;\left[n,m\right]$, where m=1,2,…,M denotes the fast-time samples in the range direction, and n=1,2,…,N denotes the index of received frames in the slow-time dimension.
2. The same MTI filter used in Equations (7) and (8) produces the clutter-suppressed signal $D_{FMCW-MTI}\left[n,m\right]$. The human range bin for each row of $D_{FMCW-MTI}\left[n,m\right]$ is determined by finding the maximum variance point with a slow-time slide window, and complex values of human points for each frame are saved as $V_{FMCW-MTI}\left[n\right]$.
3. The unwrapped the phase information of the vital signal $V_{FMCW}\left[n\right]$ is extracted, and the unwrapped phase signal is denoted as $\phi_{FMCW}\left[n\right]$.
  (16) $$\phi_{FMCW}\left[n\right]=\mathrm{unwrap}\left[\tan^{-1}\frac{Q[n]}{I[n]}\right]$$
  where Q[n] and I[n] are the imaginary and real parts of the vital signal $V_{FMCW}\left[n\right]$. The phase of the subject in the slow-time index is calculated by using $\tan^{-1}\left(.\right)$, and the phase is wrapped in [−π,π]. According to the movement of the subject, the phase can go beyond the limit of ±π. The phase unwrapping procedure is described in [28].
4. FFT spectral analysis was applied to $\phi_{FMCW}\left[n\right]$ for respiration rate and heartbeat rate.

**Figure 3:**
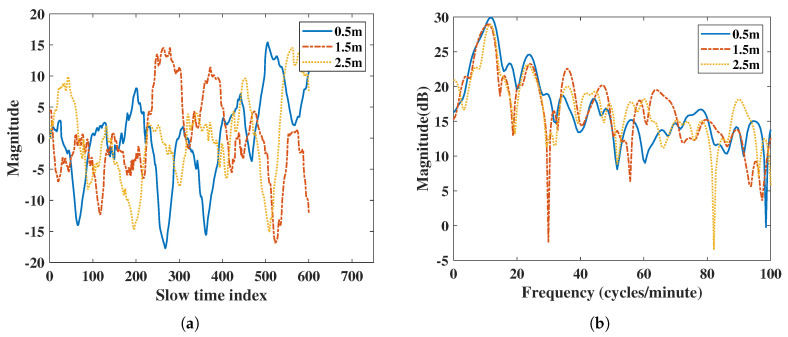
Vital signs extracted using FMCW radar at 0.5 m, 1.5 m, and 2.5 m: (**a**) vital signs extracted by FMCW radar in the time domain, (**b**) vital signs extracted by FMCW radar in the frequency domain.

Compared to IR-UWB radars, FMCW radars do not show significant magnitude differences in their vital signs at different distances in Figure 3. In order to make a fair comparison with the IR-UWB radar, the vital signs amplitude attenuation caused by the increase in distance of IR-UWB radar must be considered, and a normalized vital signs pattern between the two radars is required.

## 3. Experimental Setup

**Figure 4:**
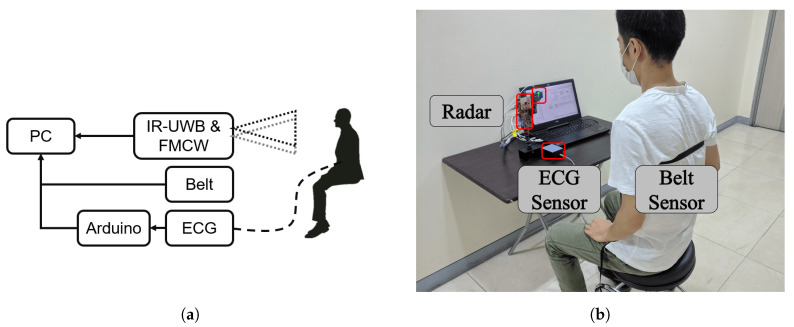
(**a**) Block dirgram of setup. (**b**) Experimental setup.

In this section, we describe the experimental setup to compare IR-UWB and FMCW radar vital signs. The block diagram of the experiment setup is shown in Figure 4. The IR-UWB and FMCW radars were placed at the same height relative to the subject, and the belt was tightened at the chest. The ECG sensor collects data using arduino, and the analog data are converted to digital. All sensors collected the data at the same start time.

To do fair comparison, we set the parameters of each radar as close as possible to each other, such as bandwidth, frame rate, and maximum range. The detailed parameters of IR-UWB are listed in Table 1, and FMCW parameters are listed in Table 2. The bandwidth of IR-UWB determines the range resolution [29] of the signal. The range resolution of FMCW radar was also determined by the sweep bandwidth. The bandwidth at different center frequencies differs by how wide the band of the system is. The bandwidth determined by the antenna design should match the bandwidth. Generally, in an IR-UWB radar system, a shorter pulse transmitted means the system occupied a wider bandwidth. However, for FMCW radar, due to the high center frequency and relative narrow bandwidth, the transmitted signal is not a wide-band signal. The bandwidth of IR-UWB radar cannot be changed, unlike FMCW radar, and in this work we set the the same bandwidths of FMCW and IR-UWB radars at 1.5 GHz. The frame rate of each radar was set to 20 FPS. When monitoring infant vital signs, the researcher set the FPS up to 40 [5].

The ADC sampling rate requirement of IR-UWB radar is higher than that of FMCW radar. The X4 chipset in IR-UWB radar uses 1-bit ADCs at 23.328 GHz sampling rate. The ADC of FMCW does not convert the received high-frequency chirp signal directly; instead, the immediate signal is the result of mixing by the received chirp signal and transmitted signal. Thus, the FMCW radar can operate with a lower ADC sampling rate than IR-UWB radar. Furthermore, the immediate signal bandwidth of FMCW is limited by the ADC sampling rate. In other words, the maximum range of FMCW is determined by the ADC sampling rate. To set the same maximum range with IR-UWB radar, we set the ADC sampling rate of FMCW at 5.5 MHz.

**Figure 5:**
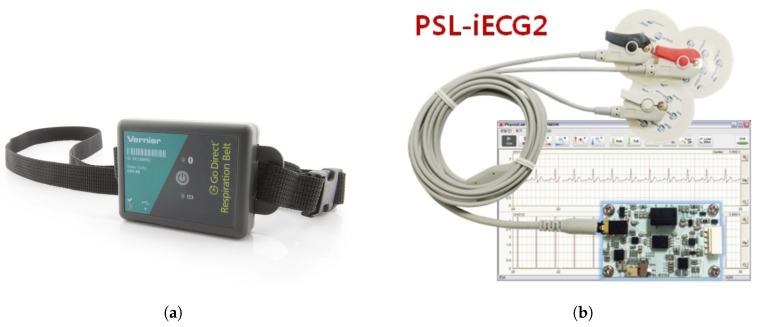
(**a**) Reference respiration belt sensor, (**b**) Reference ECG sensor module.

In order to compare the results from each scenario, we used the respiration belt and ECG sensor module as benchmark devices. In Figure 5a, the reference sensor used for the respiration rate is a respiration belt (GDX-RB, Vernier, Beaverton, OR, USA). This sensor measures the tension of chest vibrations during breathing. The force data were exported, and the frequency spectral analysis was conducted. Meanwhile, the ECG sensor module (PSL-iECG2, PhysioLab Co., Ltd., Busan, Korea) used in Figure 5b was connected to an Arduino MCU board to read the voltage during measurements. Then, a MATLAB script was used to read the digital data from the ECG board. Finally, we conducted the frequency spectral analysis with same filter used in the radar FFT spectral analysis.

In this work, we collected vital sign data for multiple scenarios from each sensor during the same measuring time. The data were recorded for 30 s for each scenario, except when comparing orientations. The accuracy ratio of radar measurements to the reference sensor is defined as follows:

(17) $$\mathrm{Ratio}=\frac{V_{M}}{V_{Ref}}\times 100\%$$

$V_{M}$ is the result of radar measurement. $V_{Ref}$ is the result of the reference sensor. The accuracy ratio is better the closer it is to 100%; ratios under or over 100% are errors.

In order to show the ability of each radar to detect vital signs, we measured the ratio of vital signs to noise and clutter. The frequency domain SNR [20] can be described as the ratio of the vital sign’s power to the other powers such as noise, clutter, or harmonics.

(18) $$\mathrm{SNR}=10\log_{10}\frac{\int_{f_{P-B}}^{f_{P+B}}\left|P_{x}\left(f\right)\right|df}{\int_{V_{L}}^{V_{H}}\left|P_{x}\left(f\right)\right|df-\int_{f_{P}-B}^{f_{P+B}}\left|P_{x}\left(f\right)\right|df}$$

$V_{L}$ and $V_{H}$ are the frequency ranges of respiration rate and heartbeat. $f_{p}$ is the peak index bin, $P_{x}\left(f\right)$ is the frequency spectrum of vital signs, and *B* is defined as the peak range.

**Figure 6:**
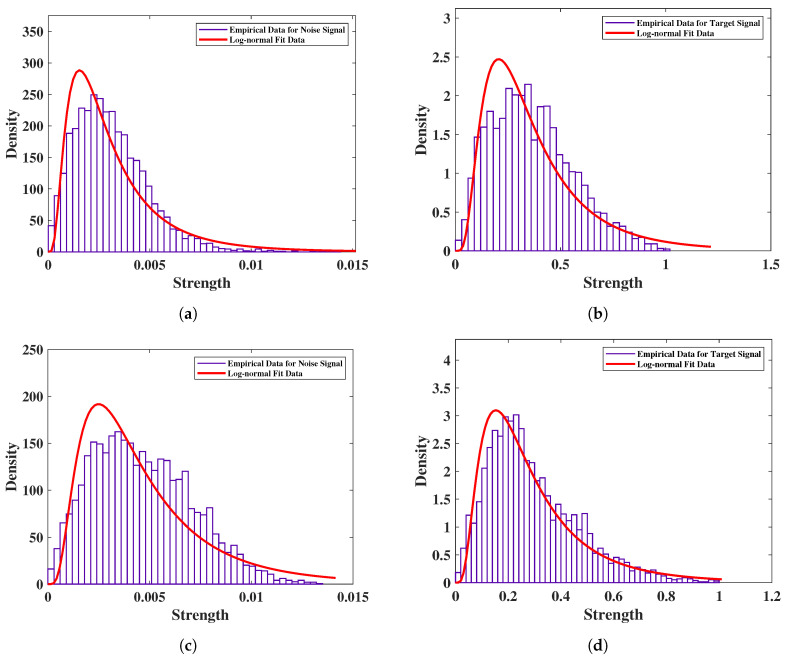
Noise and subject signal distribution of IR-UWB and FMCW radars with one subject of 1.5 m distance in and indoor open space: (**a**) distribution and fitting of noise by IR-UWB radar, (**b**) distribution and fitting of subject by IR-UWB radar, (**c**) distribution and fitting of noise by FMCW radar, and (**d**) distribution and fitting of subject by FMCW radar.

In order to verify the performance of the two radars in different operational scenarios, we performed a statistical analysis of the noise and subject signals before measuring vital signs. The statistical distribution of the received data was analyzed with normalized amplitudes, and empirical data with the log-normal fitting results were compared. Figure 6 shows the comparison of statistical data with the fitting results of the noise and subject data. From the fitting results, the noise signal and the subject signal conformed to a log-normal distribution. The mean (μ) and standard deviation (σ) of each distribution are listed in Table 3 for a total of three cases designed with one subject in an indoor open space at a distance of 1.5 m, one subject surrounded with clutter such as chairs and tables at a distance of 1 m, and one subject in front of the radar at a distance of 1 m with two layers of bricks. From the results in the table, the noise of IR-UWB was lower than that of FMCW radar, and the standard deviation was smaller in each scenario. In addition, IR-UWB had a higher target signal amplitude than FMCW radar did. Accroding to the attenuation in [27], the radar signal in the 60 GHz frequency band had a higher attenuation than the IR-UWB in X-band frequency did. Due to attenuation, the noise of FMCW was higher than that of IR-UWB radar in the case of the double bricks scenario. The $SNR_{T}$ of IR-UWB was higher than that of FMCW in time domain, which means that the vital signs were monitored with greater accuracy [26].

## 4. Experimental Results

To valid the performance of vital sign detection, we conducted the experiment in various scenarios. These scenarios were designed to compare the performances of radars, including attenuation loss in air, micro movement detection performance, penetration ability, and harmonic strength. In this experiment, data were measured with one human standing 0.5, 1.5, and 2.5 m away from the radar. The data were collected by IR-UWB, FMCW, and reference sensors at the same time. The frame rate of radars was set to 20 Hz. The radar was mounted horizontally at human chest height. The experimental environment is shown in Figure 4.

### 4.1. Distance-Based Comparison

In Figure 2, the IR-UWB results show vital signs were extracted at different distance. Due to the attenuation at different distances, the power of the vital sign signal was attenuated when the distance was far. The power of vital signs decreased about 13 dB when the distance was from 0.5 m to 2.5 m for IR-UWB. In the IR-UWB case, the algorithm measured the amplitude of estimated human point. The amplitude can be different at different distances, angles, and movement strengths. For the FMCW case, vital sign movements were extracted using phase information of the range bin in which the target was in, not by the amplitude. So, the output of the vital sign movement was not attenuated due to distance. This was different from IR-UWB radar in extracting the vital sign from a point in the algorithm. Therefore, the vital sign power of FMCW at different distances was not extensively altered, such as with IR-UWB radar. In order to compare FMCW measurements with IR-UWB measurements, the vital signs of each radar were rescaled.

**Figure 7:**
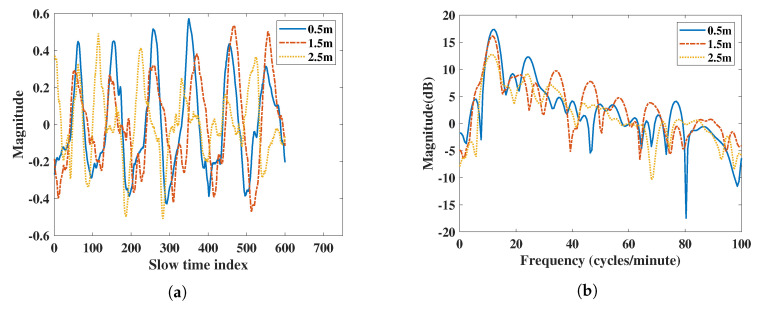

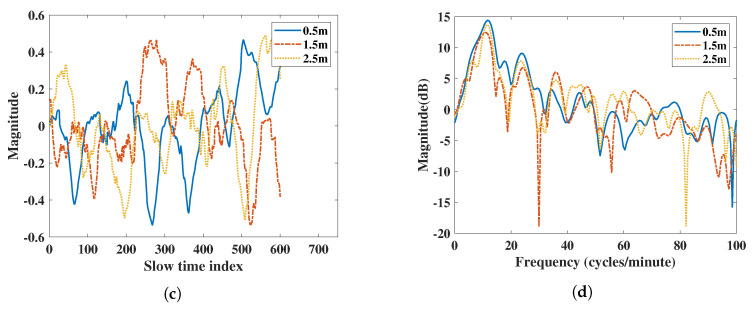
Vital signs extracted using FMCW radar at 0.5 m, 1.5 m, and 2.5 m: (**a**) vital signs extracted by IR-UWB radar in the time domain, (**b**) vital signs extracted by IR-UWB radar in the frequency domain, (**c**) vital signs extracted by FMCW radar in the time domain, (**d**) vital signs extracted by FMCW radar in the frequency domain.

From the frequency domain comparison in Figure 7b,d, the IR-UWB radar had a higher respiration power than FMCW did for 0.5 m and 1.5 m measurements. However, the FMCW was 1 dB higher than IR-UWB radar was at 2.5 m. The accuracy ratio and SNR are listed in Table 4. The RR SNR of IR-UWB was higher when the subject was closer to the radar, while the HR was also higher than that of the FMCW radar. The accuracy ratios of the two radars were similar, except the FMCW had an RR error of 0.6 (cycles/minute) at 1.5 m. Therefore, in the distance-based comparison, the performance of IR-UWB was better than that for FMCW.

### 4.2. Carotid Pulse Detection Comparison

**Figure 8:**
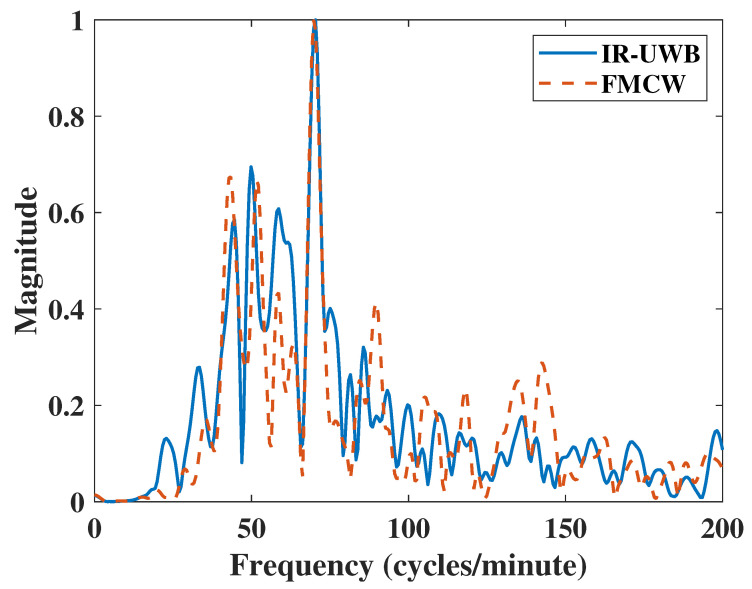
HR Results of Carotid Pulse measurements.

This experiment measured heart rate by focusing radar on a subject’s neck, where blood flowing through the carotid artery causes small variations in skin. The algorithm extracted the heartbeat by measuring the small movements of the skin. This measurement has been researched before [30,31]. The experiment was performed at a close distance of about 30 cm for 30 s. The result in Figure 8 shows that both IR-UWB and FMCW radars had similar accuracy ratios. However, there was a difference in the frequency domain; the result of IR-UWB radar had a clutter component at point 58. Furthermore, IR-UWB had two harmonic components at points 49.85 and 99.7, which may add complexity to the algorithm and reject the harmonic frequency components. In some cases, harmonics can affect the final result of the RR or HR search algorithm, resulting in significant measurement errors. Table 5 shows FMCW had a 1.2 dB higher SNR than IR-UWB did. Finally, the FMCW radar had better detection capabilities than IR-UWB radar did when measuring the carotid pulse.

### 4.3. Harmonic Comparison

**Figure 9:**
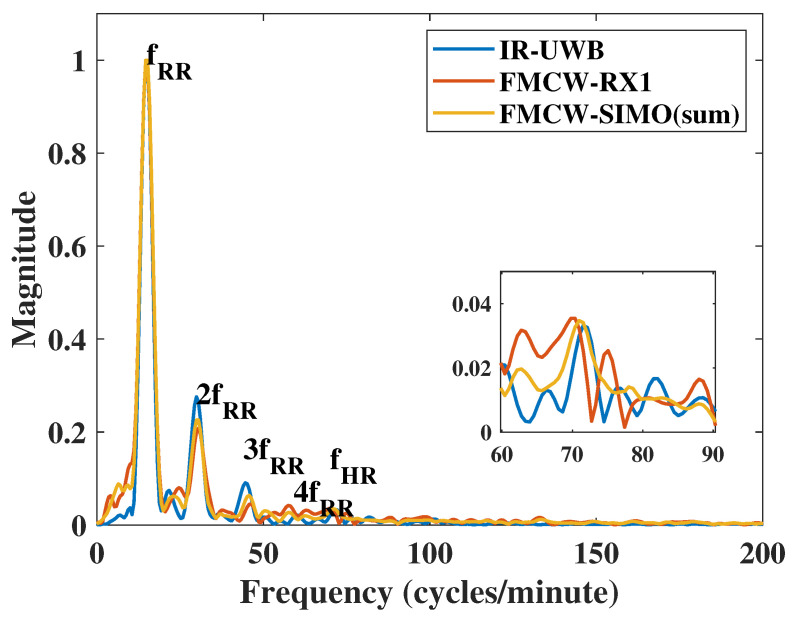
Measurement results with harmonic.

In this case, we adjusted the respiration to make the RR harmonic closer to HR. The vital signs in the frequency domain showed certain harmonic components of both IR-UWB and FMCW radars in Figure 9. The HR was close to the 5th harmonic of RR. For respiration rate, the two radars had the same accuracy ratio, but the SNR of IR-UWB was 4 dB higher than that of FMCW radar. Due to the multiple harmonics and noise, FMCW results showed a lower SNR and accuracy ratio than the IR-UWB radar did when using 1 transmit channel and 1 receive channel. However, the TI IWR6843ISK FMCW module supports four receive channels. By using the Single-input Multi-output (SIMO) topology, the radar can measure the vital sign four times in one frame from the antenna array with a λ/2 antenna spacing. According to the merge Equation (19), $P_{x}{\left(f\right)}_{i}$ is the frequency spectrum signal, *K* is the number of total receive channels, $w_{i}$ is the weight when signals merge, and $P_{merge}\left(f\right)$ is the merged signal. Here, we compared two merge methods: one is $w_{i}=1$, which means to sum each channel, and the other weight was formed in Equation (20), which follows the SNR of each channel, where $SNR_{i}$ is the SNR of each channel. After merging the results of each channel, the accuracy improved compared to using one receiving channel. From the RR results in Table 6, the SNR of FMCW improved slightly. However, in the HR results in Table 7, the SNR increased by 1.8 dB, which was higher than that of the IR-UWB radar. This is the advantage of FMCW SIMO hardware, which has a simpler structure than IR-UWB radar does. In the case of multiple harmonics, the use of multiple channels can improve the detection accuracy and SNR, especially for heartbeat detection.

(19) $$P_{merge}\;\left(f\right)\;=\;\frac{1}{K}\sum_{i=1}^{K}w_{i}P_{x}\;{\left(f\right)}_{i}$$

(20) $$w_{i}\;=\;\frac{SNR_{i}}{\max\;\left(SNR\right)}$$

### 4.4. Obstacle Penetration Comparison

Obstacle penetration when detecting humans during rescue responses using FMCW and IR-UWB radar has been studied before [32,33]. Due to the different center frequency, attenuation of the transmitted signal is also different. However, we compared the accuracy of vital signs and the SNR in the frequency domain.

**Figure 10:**
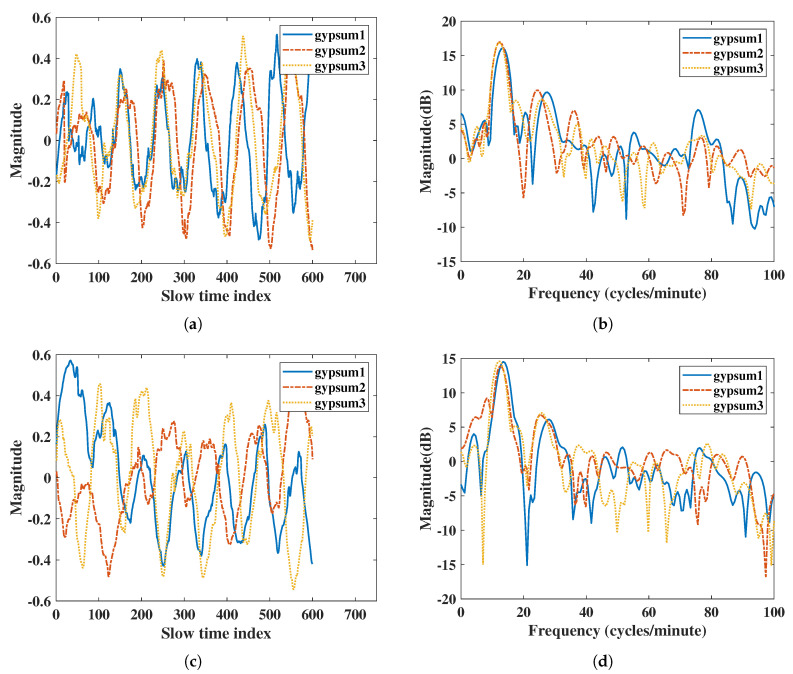
Results of penetration comparison with multiple gypsum boards: (**a**) vital signs extracted by IR-UWB radar in time domain, (**b**) vital signs extracted by IR-UWB radar in the frequency domain, (**c**) vital signs extracted by FMCW radar in the time domain, (**d**) vital signs extracted by FMCW radar in the frequency domain.

The obstacle penetration was compared by using the most commonly used construction materials. Here, we placed multiple layers of gypsum boards and multiple layers of bricks in front of the radar, and the subject was 1 m away from the radar. The single-layer gypsum board size was 200 mm × 200 mm × 10 mm, and the single brick size was 190 mm × 90 mm × 57 mm. IR-UWB extracted the vital sign signals by using the amplitude of the estimation point on the subject. The attenuation of the radar signal was large under multiple layers of obstacles. To compare the vital sign detection capabilities and power in fair conditions, we rescaled the vital sign signals extracted from both radars. The accuracy ratios of RR and HR were similar, but IR-UWB had a higher SNR than IR-UWB did in both RR and HR, Table 8. From Figure 10b,d in the frequency domain, it is clear that the peak width of IR-UWB was narrower than that of FMCW radar near the 10–20 on the x-axis where the respiration rate was measured. FMCW had a noise peak at 8 (cycles/minute) for two layers of gypsum boards, which result in a lower SNR than that for IR-UWB. Finally, the IR-UWB radar has lower noise and harmonic components than FMCW radar did.

**Figure 11:**
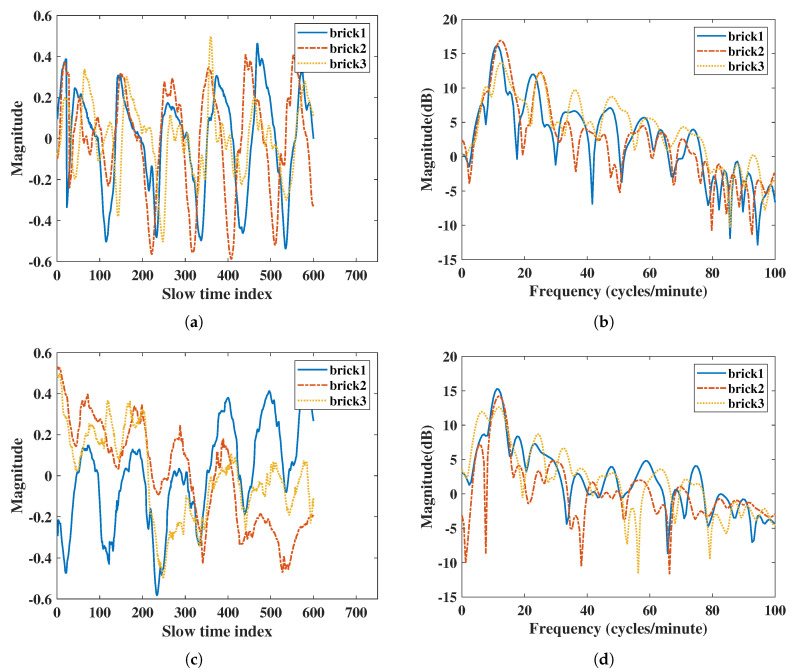
Results of penetration comparisons with multiple bricks: (**a**) vital signs extracted by IR-UWB radar in the time domain, (**b**) vital signs extracted by IR-UWB radar in the frequency domain, (**c**) vital signs extracted by FMCW radar in the time domain, (**d**) vital signs extracted by FMCW radar in the frequency domain.

For the brick penetration experiment, IR-UWB had a better SNR than FMCW radar did both in RR and HR (Table 9), similar to the gypsum board penetration comparison. The IR-UWB also had a better accuracy ratio than FMCW radar did. The FMCW had almost 5% error in RR measurements in double- and triple-layer brick comparisons, while IR-UWB was almost same as the reference belt sensor. For HR measurements, as the attenuation increased with multiple-layer bricks the accuracy ratio decreased. The FMCW had an error of up to 7.4%, and the IR-UWB had an error of up to 6.6%. There was also a noise frequency of FMCW at 8 (cycles/minute) for the triple-layer bricks comparison in Figure 11d. Generally, the IR-UWB had a better SNR and accuracy ratio than FMCW radar did when comparing penetration.

### 4.5. Orientation-Based Comparison

Since the radar system has noncontact vital sign measurement capabilities, radar has been recommended as a promising technology for monitoring vital signs in ambient assisted living (AAL) applications [34,35,36]. In a typical AAL application, monitoring of vital signs should be continuous and independent of the patient’s daily activities. However, if the patient’s orientation is not aligned with the radar, the accuracy of the vital sign measurements will be compromised. In order to find the best measurement orientation with maximum movement for a patient, the chest shift measurements performed by [37] indicated that the movement of the thorax was 4.33 mm on the front side, 1.21 mm on the lateral side, and 1.71 mm on the dorsal side. However, in real situations one’s orientation when facing the radar is not always known. There is a need to experimentally access the radar’s capabilities to measure vital signs in all four orientations.

**Figure 12:**
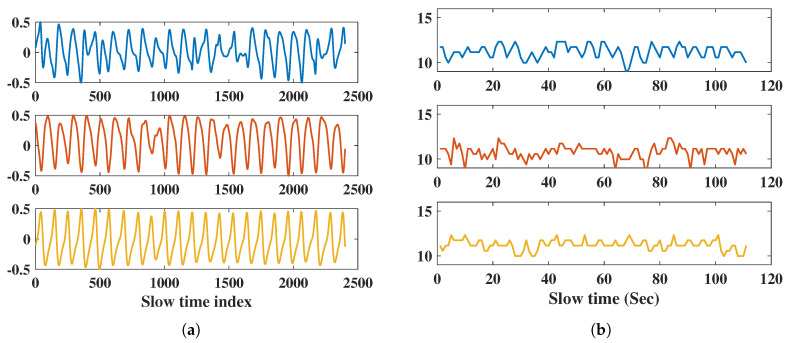
RR Measurement results of front side; (**a**) respiration signal in the time domain with IR-UWB, FMCW, and reference belt, (**b**) respiration rate of 110 s with IR-UWB, FMCW, and reference belt.

In this comparison, we collected data over 120 s for four orientations of a subject 1 m away, such as front, left (90${}^{\circ }$), right (90${}^{\circ }$), and back. We compared the real-time results of RR and HR for every second with a sliding window of 10 s, and overlap of 1 s. The time domain respiration signal and RR of the front side are shown in Figure 12. From the RMSE [38] results for each orientation in Table 10, the back side had the maximum error of RR, and the right side had the maximum error of HR. The IR-UWB radar had a lower RMSE for RR and HR on the right side than FMCW did. For the front and left sides, both radars had similar results. According to the RMSE results, IR-UWB is more accurate than FMCW.

### 4.6. Anti-Interference Comparison

In almost all typical applications, mutual interference is an issue we need to avoid. With the use of FMCW in ADAS, mutual interference between FMCW radars has been researched [39,40]. In this work, the mutual interference when using two radars was compared, considering that two or more radars can be used in an indoor environment.

**Figure 13:**
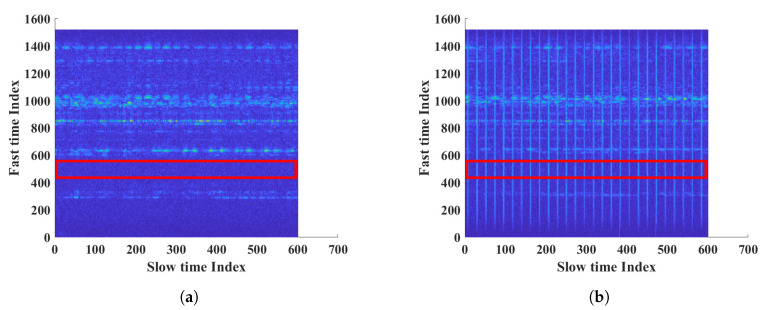
Measurement results of FMCW radar; (**a**) measurement without the FMCW interference, (**b**) measurement with the FMCW interference.

**Figure 14:**
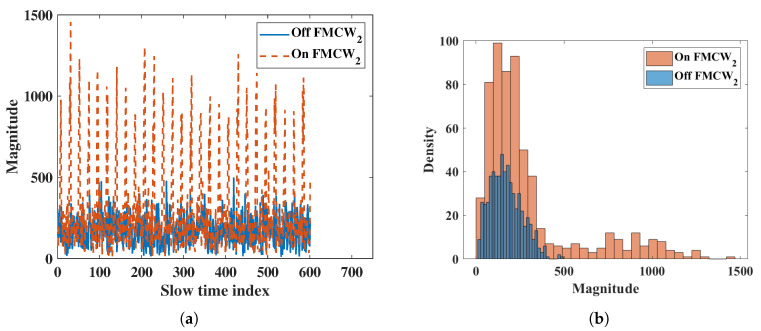
Interference results of FMCW; (**a**) slow time plot of noise at fast time index 500, (**b**) histogram of noise.

The interference radar was placed 2.5 m in front of the FMCW radar. First, we measured the noise level when the interference radar was turned on and off, without the subject. Then, we measured with the subject. Figure 13a shows measurements without the interference FMCW radar. However, the results showed large periodic noise when the interference FMCW radar was on. The normal noise and interference noise at a fast time index of 500 are shown in Figure 14a. The histogram in Figure 14b shows that the noise with interference had almost twice the power than normal. The numeric results showed a mean of 170 and standard deviation of 89.72 for normal noise and a mean of 290 and standard deviation of 282.40 for the noise with interference. As the noise power and variation increased, the SNR of the target decreased.

Vital signs with interference were measured on a subject at a distance of 1.5 m from the radar, and the interference radar was placed 1 m behind the subject. For the vital sign measurements in Table 11, the respiration rate had the same accuracy and a 2 dB lower SNR when interference was on. Meanwhile, there was 4.5% error for the heartbeat rate when interference was on. The results suggest that multiple FMCW radars should not be used in indoor environments, otherwise HR measurements will be inaccurate.

The interference with IR-UWB radar was measure. One advantage of pulsed radar is the staggered PRF [41], where the time between each coherent pulse transmission and sample event changes slightly. Thus, mutual interference can be avoided. The IR-UWB was placed in the same location as the FMCW radar. From Table 12, there were no significant errors in accuracy of RR and HR with interference radar on. Finally, multiple IR-UWB radars can be used in indoor environments without significant RR and HR errors.

### 4.7. Heavy Clutter Measurement Comparison

Monitoring vital signs in real life is more complex than it is in the laboratory, including issues such as multipaths and nonstationary clutter. To simulate the real-use case environment, we also performed measurements in a heavily cluttered environment with multiple reflections. The subject was 1 m away from the radar and surrounded by clutter components such as tables and chairs. From Table 13, both radars showed the same accuracy, but the IR-UWB radar had a higher SNR than FMCW radar did in RR and HR. Therefore, IR-UWB should be the first choice for monitoring vital signs in complex environments.

## 5. Discussion

**Figure 15:**
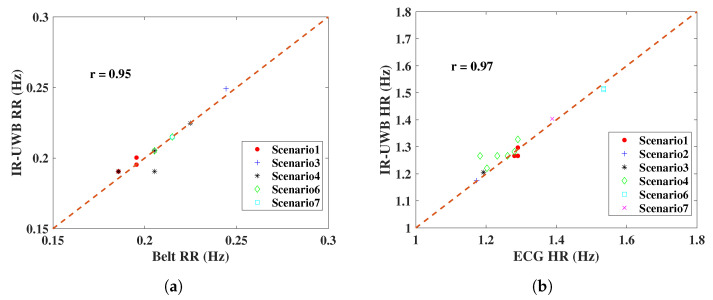

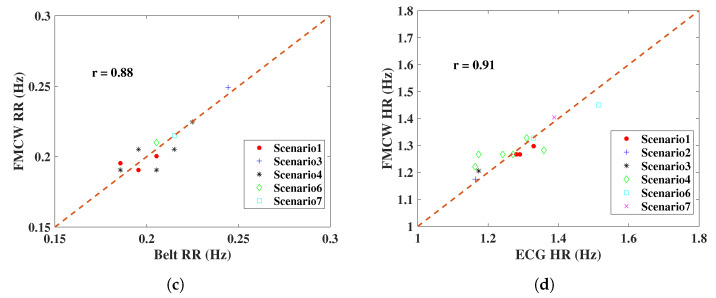
RR and HR results of radar comparisons with reference sensors in the designed scenarios; (**a**) RR comparison of IR-UWB radar, (**b**) HR comparison of IR-UWB radar, (**c**) RR comparison of FMCW radar, (**d**) HR comparison of FMCW radar. The correlation coefficient *r* is on the upper left.

**Figure 16:**
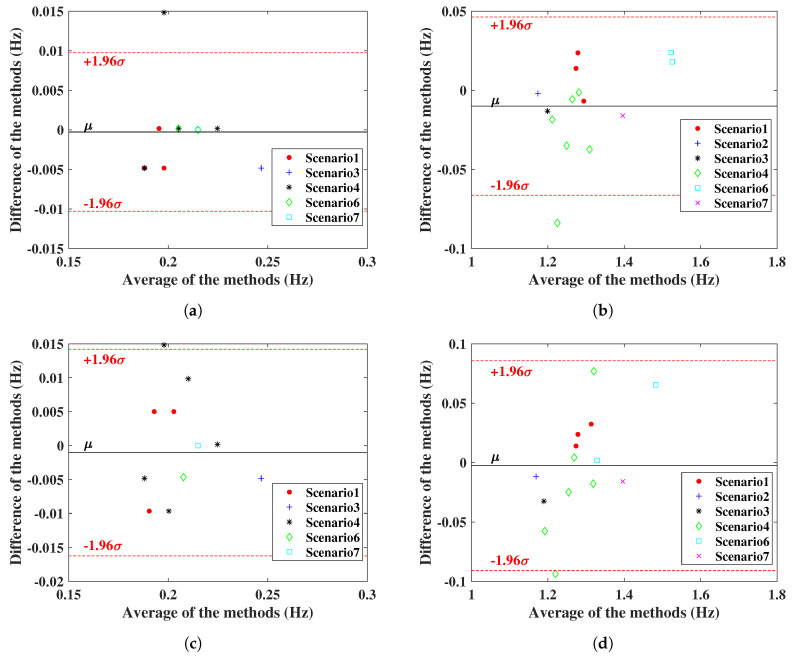
Bland–Altman comparison; (**a**) RR comparison of IR-UWB radar with respiration belt, (**b**) HR comparison of IR-UWB radar with ECG sensor, (**c**) RR comparison of FMCW radar with respiration belt, (**d**) HR comparison of FMCW radar with ECG sensor. The red lines define the limits of the confidence interval (*μ* ± 1.96*σ*), and the black line refers to the mean (*μ*) in the figures.

Experimental results of monitoring vital signs using IR-UWB and FMCW radars in different scenarios have been presented. To test the similarity of radar measurements and reference sensors, one useful parameter is Pearson’s correlation coefficient, with an interval of $\left[-1,1\right]$ [28]. If the coefficient has a value of 1, it means the measurements with the reference are completely correlated; on the contrary, 0 means they are uncorrelated. From Figure 15, the radar measurement and reference results are plotted, and the correlation coefficient *r* is on the upper left. IR-UWB showed a higher correlation with reference sensors both for RR and HR. The Bland–Altman analysis method was also applied to the data. This technique is used to compare two measurement methods, plotting the difference between radar measurement and reference against the average of the two measurements, to verify whether the measurement is significantly different or not. Supposing the data follow a Gaussian distribution, the upper and lower 95% confidence limit is ±1.96σ, where σ is the standard deviation. From Figure 16, the 95% confidence limit of IR-UWB was lower than that of FMCW radar, with a lower standard deviation. However, there was an outlier for each radar in the obstacle penetration scenario. As mentioned earlier, a lower SNR will affect the accuracy of vital signs. Finally, both radar systems are capable of monitoring RR and HR in close range. However, considering all scenarios, measurements with IR-UWB radar were closer to the reference than they were with FMCW radar. FMCW radar also has its own advantages, and the measurement results can be enhanced by combining all receiving channels with MIMO topologies in FMCW. In addition, the DOA of the target can be estimated according to the signal delay in a linear antenna array. In contrast, interference is a drawback that may limit the use of multiple FMCW radars.

## 6. Conclusions

In recent years, researchers have presented several new and interesting methods to measure vital signs in noncontact or rescue purpose scenarios using radar. In this paper, IR-UWB and FMCW radar techniques are briefly reviewed, discussed, and compared in various scenarios under the same conditions. For almost all comparison scenarios, the IR-UWB radar had better accuracy ratios and higher SNRs than the FMCW radar did. Conversely, with its simple hardware structure, the FMCW radar can also deal with multi-harmonic situations by combining the vital sign results for each channel. Due to the advantages of pulse penetration and the lower frequency band, the IR-UWB radar had a better accuracy and higher SNR than the 60 GHz FMCW radar in the obstacle penetration comparison. Compared to IR-UWB pulses, normal FMCW chirps have long durations and were not coded. It is better to use only one FMCW radar in an indoor environment, otherwise significant errors may result due to mutual chirp interference. In addition, in a heavily cluttered environment, both radars had the same accuracy, but the SNR of IR-UWB was higher than that of FMCW radar. According to the designed scenarios, the IR-UWB radar had more advantages than FMCW radar did, with a higher accuracy and higher SNR, allowing vital signs to be monitored in a variety of environments.

## Figures and Tables

**Table 1:** IR-UWB Radar Features.

IR-UWB System Parameters	Value
IR-UWB chipset	Novelda X4
Center frequency	8.7 GHz
Bandwidth (−10 dB)	1.5 GHz
ADC sampling rate	23.328 GHz
Peak pulse output power	6.3 dBm
Pulse repetition frequency	40.5 MHz
Slow-time sampling frequency	20 FPS

**Table 2:** FMCW Radar Features.

FMCW System Parameters	Value
FMCW chipset	TI IWR6843ISK
Starting frequency	60 GHz
Sweep bandwidth	1.5 GHz
ADC sampling rate	5.5 MHz
Transmitter output power	12 dBm
Chirp slope	32.251 MHz/us
Slow-time sampling frequency	20 FPS

**Table 3:** Statistical Analysis of Noise and Subject Signal.

		Indoor Open Space			Heavy Clutter			Double Brick		
		Mean (μ)	Std (σ)	$\mathrm{SNR}{}_{T}$ (dB)	Mean (μ)	Std (σ)	$\mathrm{SNR}{}_{T}$ (dB)	Mean (μ)	Std (σ)	$\mathrm{SNR}{}_{T}$ (dB)
IR-UWB	Noise	0.0031	0.0017	21.0	0.0061	0.0029	18.48	0.0029	0.0018	21.50
	Target	0.39	0.19		0.43	0.20		0.41	0.18	
FMCW	Noise	0.0046	0.0024	18.0	0.0062	0.0036	17.26	0.067	0.034	10.56
	Target	0.29	0.17		0.33	0.16		0.33	0.20	

**Table 4:** RR and HR Results from the distance-based comparison.

		0.5 m			1.5 m			2.5 m		
		Value	Ratio (%)	SNR (dB)	Value	Ratio (%)	SNR (dB)	Value	Ratio (%)	SNR (dB)
RR	IR-UWB	11.73	97.6	7.34	11.73	100.1	5.44	11.14	97.5	4.4
	FMCW	12.32	102.5	6.8	11.14	95.1	6.75	11.73	102.6	6.1
	Belt	12.02			11.72			11.43		
HR	IR-UWB	76.83	101.1	−3.37	77.42	99.5	−5.15	77.42	101.7	−5.59
	FMCW	76.83	101.1	−5.47	79.77	102.5	−6.1	77.42	101.7	−6.88
	ECG	76.0			77.83			76.0		

**Table 5:** HR Results of Carotid Pulse measurements.

	HR	Ratio (%)	SNR (dB)
IR-UWB	70.38	99.8	−2.80
FMCW	69.79	99.0	−1.63
ECG	70.5		

**Table 6:** RR Results of measurement with harmonics.

	RR	Ratio (%)	SNR (dB)
IR-UWB	14.66	98.1	11.01
FMCW-RX1	14.66	98.1	7.43
FMCW-SIMO (sum)	14.66	98.1	7.70
FMCW-SIMO (SNR)	14.66	98.1	7.91
Belt	14.95		

**Table 7:** HR Results of measurement with harmonics.

	HR	Ratio (%)	SNR (dB)
IR-UWB	71.55	98.9	−3.74
FMCW-RX1	70.38	97.9	−5.18
FMCW-SIMO (sum)	70.79	98.1	−3.80
FMCW-SIMO (SNR)	71.55	98.9	−3.30
ECG	72.34		

**Table 8:** RR and HR Results of Penetration Comparison with Multiple Gypsum Boards.

		Single Gypsum Board			Double Gypsum Board			Triple Gypsum Board		
		Value	Ratio (%)	SNR (dB)	Value	Ratio (%)	SNR (dB)	Value	Ratio (%)	SNR (dB)
RR	IR-UWB	13.49	100.1	8.17	12.32	100.1	10.5	12.32	107.8	7.48
	FMCW	13.49	100.1	8.12	12.9	104.8	5.32	12.32	107.8	8.37
	Belt	13.48			12.31			11.43		
HR	IR-UWB	75.66	99.6	−0.78	76.83	99.9	−5.45	77.42	97.2	−3.96
	FMCW	76.25	100.3	−3.47	81.52	106.0	−5.91	78.59	98.6	−4.05
	ECG	76.0			76.91			79.66		

**Table 9:** RR and HR Results of Penetration Comparisons with Multiple Bricks.

		Single Brick			Double Brick			Triple Brick		
		Value	Ratio (%)	SNR (dB)	Value	Ratio (%)	SNR (dB)	Value	Ratio (%)	SNR (dB)
RR	IR-UWB	11.14	97.5	7.40	12.32	100.1	9.06	12.32	100.1	3.57
	FMCW	11.14	97.5	4.83	11.73	95.3	7.46	11.73	95.3	2.44
	Belt	11.43			12.31			12.31		
HR	IR-UWB	73.9	97.2	−3.85	72.14	98.5	−5.42	70.97	93.4	−5.78
	FMCW	74.49	98.0	−3.70	69.79	95.3	−5.18	70.38	92.6	−7.26
	ECG	76.0			73.25			76.0		

**Table 10:** RMSE results of radar measurements for each orientation.

RMSE		Front	Left (90${}^{\circ }$)	Right (90${}^{\circ }$)	Back
RR	IR-UWB	1.0	1.3	1.2	3.3
	FMCW	1.1	1.5	1.7	3.1
HR	IR-UWB	3.9	3.8	4.2	3.9
	FMCW	3.8	4.0	4.4	4.17

**Table 11:** Anti-interference results of FMCW radar.

FMCW	Without Interference			With Interference		
	Value	Ratio (%)	SNR (dB)	Value	Ratio (%)	SNR (dB)
RR	12.32	97.8	8.21	12.32	97.8	6.27
Belt	12.6			12.6		
HR	79.77	100.1	−5.07	90.91	104.5	−4.87
ECG	79.66			86.99		

**Table 12:** Anti-interference results of IR-UWB radar.

IR-UWB	Without Interference			With Interference		
	Value	Ratio (%)	SNR (dB)	Value	Ratio (%)	SNR (dB)
RR	12.9	100	7.02	12.32	100.1	7.08
Belt	12.9			12.31		
HR	92.08	101.2	−4.65	92.08	101.6	−5.92
ECG	91			90.65		

**Table 13:** RR and HR results under a heavily cluttered environment.

		Value	Ratio (%)	SNR (dB)
RR	IR-UWB	12.9	100	9.50
	FMCW	12.9	100	6.74
	Belt	12.9		
HR	IR-UWB	83.28	98.9	−4.71
	FMCW	83.28	98.9	−6.21
	ECG	84.24		
